# Immunohistochemical analysis of Enolase-1 sublocalization in benign and malignant breast tumors: potential implications for tumor progression and prognosis

**DOI:** 10.3389/fonc.2025.1636394

**Published:** 2025-10-16

**Authors:** Laura de Souza Baracioli, Caroline Patini Rezende, Luadna dos Santos e Silva, Débora de Lima Alves, Daniela Farias da Nóbrega, Luiz Gustavo de Almeida Chuffa, Debora Aparecida Pires Campos Zuccari

**Affiliations:** ^1^ Laboratory of Molecular Investigation in Cancer (LMIC), Department of Molecular Biology, Faculty of Medicine of São José do Rio Preto (FAMERP), São José do Rio Preto, Brazil; ^2^ Institute of Biosciences, Letters and Exact Sciences (IBILCE), UNESP, São José do Rio Preto, Brazil; ^3^ Pat Animal Laboratory, São José do Rio Preto, Brazil; ^4^ Department of Structural and Functional Biology, Institute of Biosciences, São Paulo State University (UNESP), Botucatu, Brazil

**Keywords:** breast cancer, immunohistochemistry, enolase-1, biomarkers, ROC curve analysis

## Abstract

Breast cancer is the second most common neoplasm in women and one of the main causes of premature mortality, with a high incidence before the age of seventy. Among its histological subtypes, invasive ductal carcinoma accounts for approximately 65% to 70% of cases and is characterized by significant molecular and prognostic heterogeneity. Although some molecular subtypes benefit from targeted therapies, triple-negative carcinomas remain a considerable clinical challenge, predominantly affecting young women who often subjected to highly aggressive and not always effective conventional treatments. The identification of prognostic and predictive biomarkers is essential to optimize therapeutic choices and anticipate potential resistance mechanisms. Enolase-1 (ENO1), a glycolytic enzyme involved in cellular energy homeostasis, has been widely associated with tumor progression and metabolic adaptation in malignant neoplasms. In this study, we investigated ENO1 expression in benign and malignant breast tumors using immunohistochemistry, analyzing both the tissue distribution pattern and staining intensity. Our results suggest that ENO1 may play a predictive diagnostic role, aiding in more individualized therapeutic strategies and contributing to the advancement of precision medicine in breast cancer.

## 1 Introduction

Breast cancer (BC) is the second leading cause of cancer death among women, after lung cancer, and is a major adversary in the global health challenges scenario. Its complex pathogenesis and diverse clinical manifestations representing significant obstacles to effective diagnosis, treatment, and prevention (1, 2). The histological classification of this neoplasm reveals the marked structural heterogeneity of these tumors, which are predominantly of epithelial origin (3). Invasive ductal carcinoma (IDC), the most common histological type, accounts for approximately 65% to 70% of all diagnosed cases. It is characterized by the infiltrating behavior of breast epithelial cells with the potential to affect distant organs due to its high propensity to metastasize, and consequently associated with a worse prognosis (4, 5).

Given the importance of the BC diagnosis, which includes determining the histological type, molecular subtype, and appropriate treatment, there is a need to investigate new biomarkers. These agents must possess characteristics that allow for early diagnosis, determination of the origin of the tumor, assessment of disease extent, monitoring of therapeutic response, and early detection of recurrences (6, 7). ENO1 is an essential enzyme in the glycolytic pathway, responsible for catalyzing the conversion of 2-phospho-D-glycerate into phosphoenolpyruvate, a vital cellular mechanism that generates the energy needed for cell function and proliferation (8). However, this enzyme is also strongly expressed in a subset of tumors and is involved in the Warburg effect, a pro-tumor metabolic mechanism characterized by the predominance of aerobic glycolysis even in the presence of oxygen (9). In addition, ENO1 can perform different functions depending on its intracellular or extracellular location, reinforcing its functional versatility in the tumor microenvironment (10, 11).

According to Huang et al. (2022) (12), overexpression of ENO1 has shown significant diagnostic and prognostic value in a wide range of tumors. In turn, the proteomic study of extracellular vesicles (EVs), conducted by our research group identified the ENO1 enzyme as a candidate biomarker for the diagnosis of these tumors (13). Our results demonstrate the presence of ENO1 in all tumor samples analyzed, with a predominance in malignant tumors. The ENO1 coefficient was not statistically significant enough to be considered a predictor of prognosis; however, staging analysis inferred a higher risk of unfavorable clinical outcomes. Therefore, a relationship can be inferred between the intensity of ENO1 immunohistochemical staining and the clinical outcomes of breast cancer patients.

## 2 Materials and methods

### 2.1 Patient selection

A retrospective approach was carried out by reviewing medical records from 2013 to 2018, selecting patients with invasive ductal breast cancer from the Hospital de Base in São José do Rio Preto. The study was approved by the Ethics and Research Committee/FAMERP (protocol number 4.007.723). The medical records were thoroughly reviewed to identify cases with a confirmed diagnosis of invasive ductal carcinoma (IDC), histological grade II, and 41 patients were selected (**Table 1**). The patients had varied outcomes: some responded favorably to treatment and had remission, while others faced unfavorable outcomes, such as death or the development of recurrence/metastasis. We confirm that only patients whose death was attributed to health complications caused by cancer were included in our analysis. In addition to the patients with IDC, seven patients diagnosed with benign lesions, specifically fibroadenomas, were included in the control group. This design allowed a detailed comparative assessment of ENO1 expression profiles between patients with different clinical outcomes and those with benign lesions, providing a solid basis for interpreting the results.

**Table 1:** Data on patients with malignant and benign breast tumors were analyzed retrospectively from 2013 to 2018.

ID	Histoscore	Subtype	Ki-67	Surgery	Staging	Outcome
1.1	95.4167	Luminal A	<10%	2018	Stage IIA	Alive
2.1	88.7206	Luminal A	20% a 25%	2017	Stage IIB	Alive
3.1	96.7822	Luminal A	<10%	2018	Stage IA	Alive
4.1	91.5188	Luminal A	9%	2017	Stage IA	Alive
5.1	73.6743	Luminal A	10% a 25%	2016	Stage IA	Alive, relapse/metastasis
6.1	86.6066	Luminal A	<10%	2014	Stage IA	Death in 2023
7.1	86.9978	Luminal A	10% a 15%	2016	Stage IIIB	Death in 2021
8.2	146.3258	Luminal B	>30%	2017	Stage IIA	Death in 2022
9.2	78.8089	Luminal B	>30%	2013	Stage IIA	Death in 2018
10.2	42.3464	Luminal B	>30%	2015	Stage IIIA	Alive, relapse/metastasis
11.2	98.0764	Luminal B	50%	2015	Stage IIA	Alive
12.2	62.6363	Luminal B	20%	2016	Stage IV	Alive
13.2	79.8502	Luminal B	20%	2018	Stage IIA	Alive
14.2	116.0637	Luminal B	10% a 25%	2016	Stage IIIA	Alive, relapse/metastasis
15.2	99.6945	Luminal B	30%	2018	Stage IIA	Alive
16.2	50.33622	Luminal B	40% a 50%	2018	Stage IIB	Alive
17.2	65.4485	Luminal B	>25%	2018	Stage IIA	Alive
18.2	104.0055	Luminal B	30%	2018	Stage IB	Alive
19.2	118.2339	Luminal B	18%	2017	Stage IIA	Death in 2021
20.2	107.8642	Luminal B	>25%	2018	Stage IIIA	Death in 2021
21.2	104.89196	Luminal B	40% a 50%	2018	Stage IA	Alive
22.2	69.8232	Luminal B	40%	2018	Stage IB	Alive
23.2	67.5771	Luminal B	80% a 90%	2018	Stage IA	Alive
24.2	143.1414	Luminal B	>25%	2017	Stage IV	Death in 2018
25.2	145.1700825	Luminal B	40% a 50%	2019	Stage IV	Death in 2020
26.3	60.9861	HER2-positive	10% a 25%	2014	Stage IV	Death in 2014
27.3	46.9729	HER2-positive	40%	2017	Stage IIB	Death in 2021
28.3	63.5255	HER2-positive	40%	2018	Stage IA	Alive
29.3	65.8315	HER2-positive	30%	2018	Stage IIA	Alive
30.3	55.1915	HER2-positive	<10%	2018	Stage IIIB	Alive, relapse/metastasis
31.3	103.6907	HER2-positive	>25%	2013	Stage IIIA	Death in 2017
32.4	121.3378	Triple-negative	>30%	2014	Stage IA	Alive
33.4	113.1591	Triple-negative	95%	2018	Stage IIIC	Death in 2022
34.4	95.5023	Triple-negative	60%	2015	Stage IIA	Death in 2018
35.4	76.4999	Triple-negative	>30%	2016	Stage IA	Alive
36.4	121.0221	Triple-negative	>30%	2016	Stage IIIB	Death in 2017
37.4	39.9331	Triple-negative	90%	2013	Stage IIA	Death in 2023
38.4	93.4637	Triple-negative	30%	2016	Stage IA	Alive, relapse/metastasis
39.4	142.5616	Triple-negative	70%	2018	Stage IIIC	Death in 2019
40.4	104.5667	Triple-negative	50%	2018	Stage IIIB	Death in 2020
41.4	96.1282	Triple-negative	>25%	2016	Stage IV	Death in 2016
42.5	98.7185	Fibroadenoma	–	2023	–	Alive
43.5	39.0504	Fibroadenoma	–	2022	–	Alive
44.5	101.0852	Fibroadenoma	–	2022	–	Alive
45.5	64.2314	Fibroadenoma	–	2021	–	Alive
46.5	32.6115	Fibroadenoma	–	2021	–	Alive
47.5	42.0604	Fibroadenoma	–	2021	–	Alive
48.5	26.9689	Fibroadenoma	–	2021	–	Alive

### 2.2 Immunohistochemistry

For immunohistochemical staining, 4 μm-thick tissue sections were prepared using a microtome from the manufacturer Leica (Model 2255, Wetzlar, Germany). The slides were deparaffinized in xylene and rehydrated through a series of graded alcohols, followed by incubation in an oven at 60 °C for 3 hours. Antigen recovery was carried out using EDTA buffer (pH 7.4) for ENO1, heated to 100 °C in a steam cooker (Histo Bath, from the manufacturer Easy Path, São Paulo - SP, Brazil) for 30 minutes. Endogenous peroxidase activity was blocked using a DAKO peroxide solution for 15 minutes, and non-specific protein binding was minimized with a 3% BSA solution in PBS for 10 minutes.

The recombinant anti-ENO1 primary antibody [EPR 19758] (ab227978 - ABCAM) was used at a 1:3000 dilution and incubated overnight at 4 °C to ensure specific binding. Peroxidase (HRP)-conjugated secondary antibodies were then applied for 25 minutes. Immunoreactivity was visualized using diaminobenzidine tetrahydrochloride (DAB) as a chromogen (1 per 1000 μL of DAKO substrate) for 2 min. Finally, the slides were counterstained with hematoxylin for 2 min to improve visualization of the cell structure and mounted for analysis. All immunoreactions were accompanied by a positive control for the antibody tested (small intestine) and a negative control (antibody absent).

Breast tumor samples were obtained from the Pathology Department of Hospital de Base de São José do Rio Preto, while positive control samples for the immunohistochemistry technique were provided by the Pathology Teaching Laboratory of the Faculty of Medicine of São José do Rio Preto (FAMERP).

### 2.3 Capture of images of histopathological slides

The images were captured using a Nikon Eclipse E200 microscope equipped with a 40x magnification objective and an attached camera (Leica EC3). Five random fields from each slide, and 100 tumor cells per field were counted. Cells were classified as positive when ENO1 staining was observed in the cytoplasm, cell membrane, or nucleus, appearing as yellow to brownish-yellow granules. Samples with ≥5% positive cells were considered positive, whereas those with <5% were considered negative (14). The entire tissue area was systematically examined for image acquisition, with particular focus on tumor regions. Areas with excessive inflammation, necrosis, or features compatible with normal tissue were excluded. Selection prioritized sites with intense cell proliferation, often showing nuclear pleomorphism, cribriform patterns, and a predominance of desmoplastic reaction.

### 2.4 Analysis of immunohistochemistry results

ENO1 expression was quantified using the ImageJ software and the “Immunohistochemistry (IHC) Image Analysis” tool, which generates a histoscore (HS). This method converts the qualitative analysis of classical immunohistochemistry into a quantitative scale. The HS is based on the intensity of the staining and the percentage of stained cells, categorized as: negative (0), weak (1+), moderate (2+), and strong (3+). For each case, the HS was calculated with a potential range of 0–300 as follows: HS = (1 × % of weakly stained cells) + (2 × % of moderately stained cells) + (3 × % of strongly stained cells) (15, 16).

### 2.5 Statistical analysis

**Figure 1:**
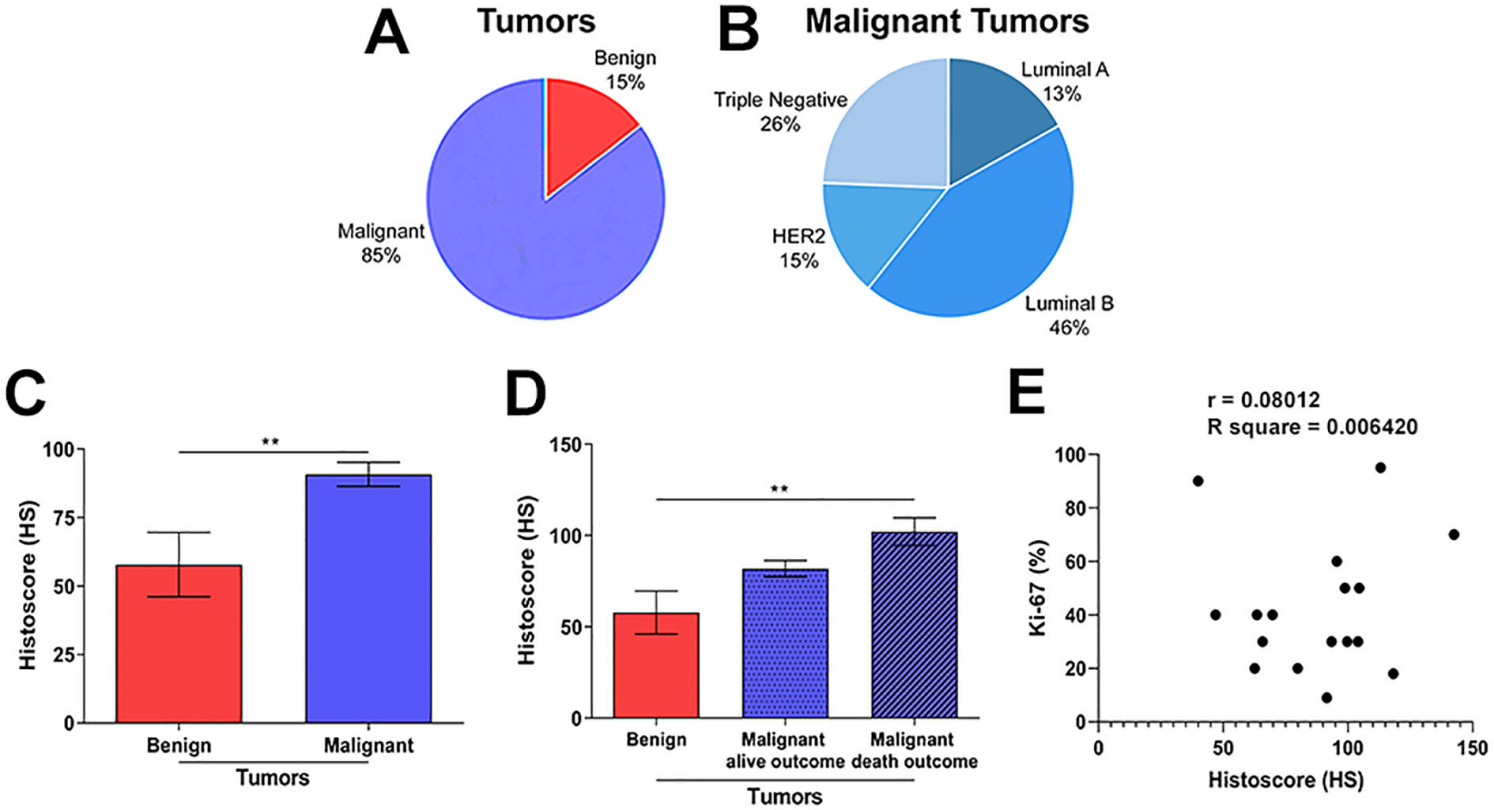
Analysis of the qualitative and quantitative distribution through the histoscore (HS) of benign and malignant tumors and their molecular subtypes. **(A)** Quantitative distribution of the different types of tumors and **(B)** the molecular subtypes of breast cancer analyzed in the study. **(C)** Difference in HS means between the samples of the groups with benign (Fibroadenoma) and malignant (Luminal A, Luminal B, HER2 overexpressed, and triple-negative) breast tumors, and **(D)** related to the outcomes of alive and death. Statistical difference: **p<0.01. **(E)** Pearson correlation was performed with the absolute values of Histoscore and KI-67 of patients with malignant breast cancer. Statistical analysis of r = 0,08012; R-squared = 0.006420 and p = 0.7598.

Statistical analyses and graphs generation were performed using GraphPad Prism (v. 8.0). The normality of residuals for quantitative variables was assessed with the Shapiro-Wilk test. Group comparisons were performed using one-way ANOVA, followed by Student’s t-tests (**Figure 1C**). Multiple comparisons were conducted with Tukey’s *post hoc* test (**Figure 1D**). To evaluate the prognostic value of ENO1, ROC curve analysis was performed in GraphPad Prism (v.8.0) and Cox linear regression was conducted in SPSS (IBM, version 24, 2014) using the Omnibus Test of Model Coefficients.

## 3 Results

### 3.1 Expression of ENO1 and its marker sites

**Figure 2:**
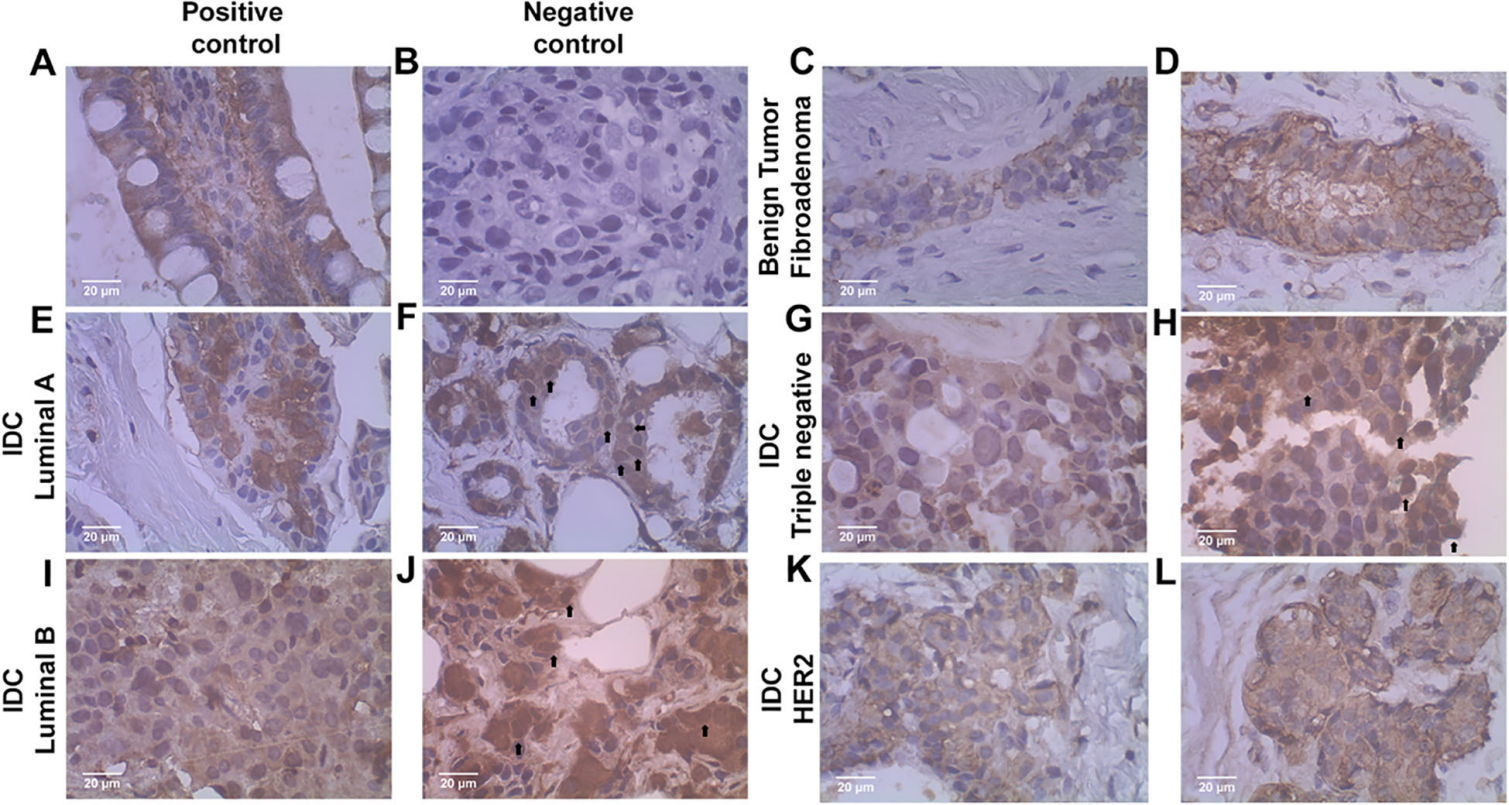
Photomicrographs of Enolase-1 (ENO1) staining sites in breast tumors. Staining of positive **(A)** and negative **(B)** controls, represented by small intestine and breast tumor sample with omission of antibody, respectively. Benign breast tumors of the fibroadenoma type with cytoplasmic staining **(C)** and cytoplasmic and membrane staining **(D)**. Malignant breast tumors, IDC, grade II, molecular subtypes: Luminal A, with cytoplasmic staining **(E)**; and cytoplasmic and nuclear staining **(F)**. Triple-negative, with cytoplasmic staining **(G)**, and cytoplasmic and nuclear staining **(H)**. Luminal B, with cytoplasmic staining **(I)**, and cytoplasmic and nuclear staining **(J)**. HER2 overexpressed, with cytoplasmic staining **(K, L)**. All nuclear stainings are indicated by the arrow.

Immunohistochemical staining was visualized by the golden-brown color, indicating the presence of the ENO1 enzyme in the cells analyzed. Predominantly cytoplasmic staining was observed, accompanied in some cases by nuclear and membrane staining, the latter always associated with cytoplasmic staining (**Figure 2**). In the photomicrographs analyzed, the staining patterns were cytoplasmic (**Figures 2A, C, E, G, I, K**), simultaneous cytoplasmic and membrane (**Figure 2D**), simultaneous cytoplasmic and nuclear (**Figures 2D, F, J**), and absence of staining due to the omission of the antibody (**Figure 2B**).

Cytoplasmic staining was predominant in all samples, regardless of whether they had a benign or malignant profile. In addition, membrane staining was observed in both tumor types, although it was more frequently associated with the malignant profile. ENO1 nuclear staining, however, was detected exclusively in samples with a malignant profile. All the samples analyzed, benign tumors (**Figures 2C, D**) and malignant tumors (**Figures 2E-L**), showed immunohistochemical staining. In the malignant tumors, the staining was more intense and diversified, while in the benign tumors, the staining predominated in the cytoplasm and membrane of the myoepithelial cells of the mammary ducts. This suggests that the respective and varied sites of staining, as well as their intensity, should be further investigated and could be of great help in characterizing the malignancy profile of the tumors and subsequent clinical management of the patients.

### 3.2 ENO1 staining intensity related to tumor type and outcome

The analysis of data from the medical records of the tumor samples from the 48 patients in the study (**Table 1**) showed that 15% of the tumors were benign and 85% were malignant (**Figure 1A**). Of the malignant tumors, 13% were of the Luminal A subtype, 46% Luminal B, 15% HER2 overexpressed, and 26% triple-negative (**Figure 1B**). Statistical analysis of patient record data showed normal distribution, confirmed by the Shapiro-Wilk test, with a 95% confidence interval (CI). Histoscores (HS) were compared between benign and malignant tumors samples (**Supplementary Material S2**), without considering patients’ clinical outcomes. An unpaired t-test revealed a significant difference between benign and malignant tumors (p=0.0035 and CI=-56.44 to -9.409), indicating that the intensity of ENO1 immunohistochemical staining is directly related to the type of breast tumor (**Figure 1C**).

Subsequently, comparisons were made between the benign tumor and malignant tumor groups, related to the outcome (alive) and the outcome (death), according to the data in **Table 1**. Between the benign (control) and malignant groups with a live outcome, no statistically significant difference was observed (**Figure 1D**). However, statistically significant differences were identified between the benign and malignant groups with a death outcome (**Figure 1D**). The mean differences between the groups were benign vs. malignant (alive outcome) was -24.04 (CI= -52.51 to 4.434); benign vs. malignant (death outcome) was -44.27 (CI=-73.66 to - 14.89); malignant (alive outcome) vs. malignant (death outcome) was -20.24 (CI= -40.99 to 0.5216). These findings suggest that ENO1 expression increases proportionally to the degree of malignancy of breast tumors, correlating with more severe outcomes.

To determine the direct relationship between ENO1 and the cell proliferation index (Ki-67) of malignant tumors, which is essential for tumor growth, a correlation analysis was performed. The data were normalized, and Pearson’s correlation was performed using the absolute values of histoscores and Ki-67 in patients with malignant breast cancer. A very weak correlation was observed between the variables (r = 0.08; R-squared = 0.006420; 95% CI = -0,4166 to 0,5400; and p = 0.7598), indicating that there is virtually no linear relationship between them (**Figure 1E**). However, the lack of statistically significant findings highlights the need for additional, more comprehensive studies to better understand the clinical and biological relevance of ENO1.

### 3.3 Evaluation of the prognostic potential of ENO1 in breast tumors

**Figure 3:**
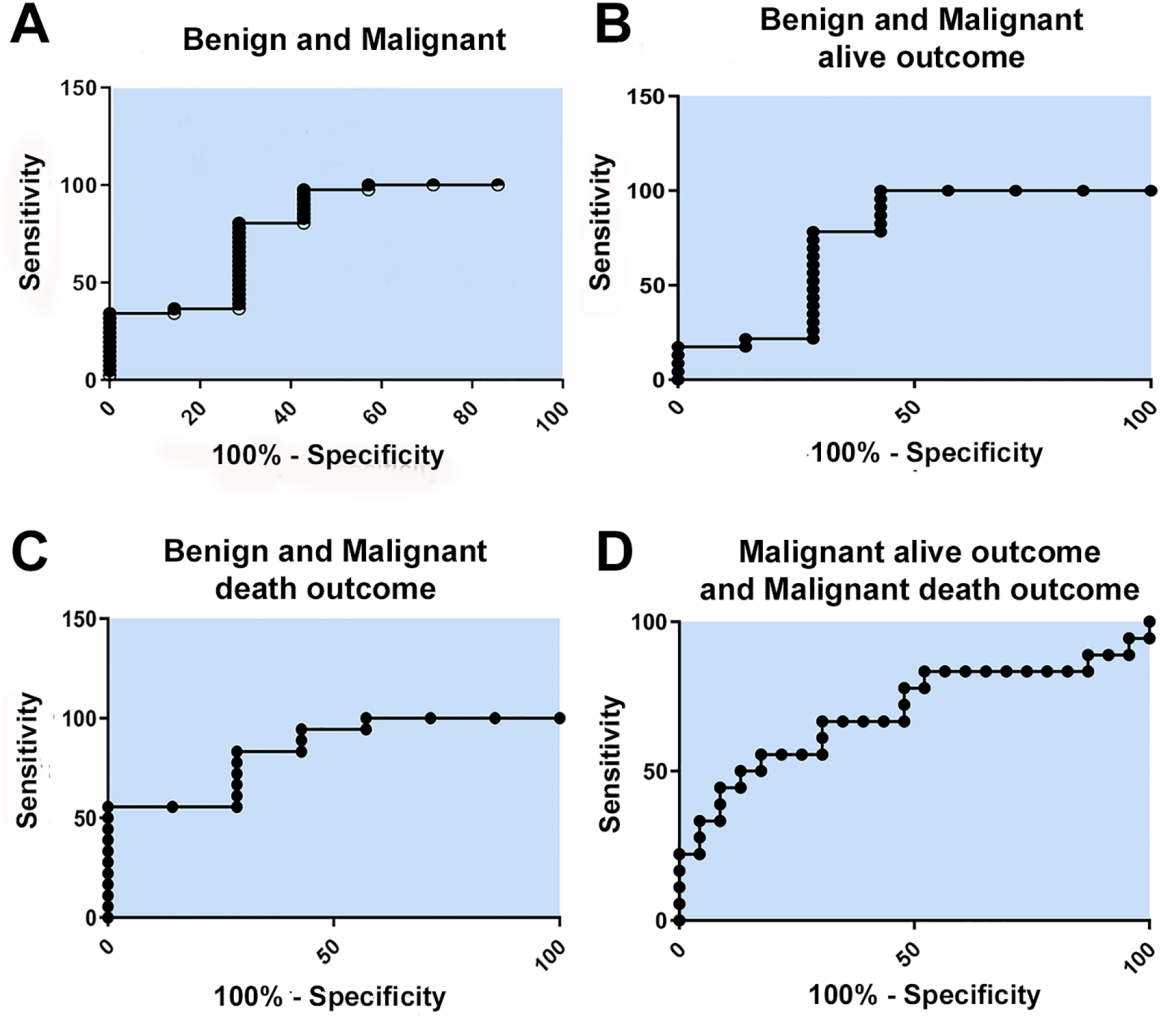
Sensitivity and specificity analysis of ENO1 staining. **(A)** ROC curve with AUC = 0.78; **(B)** ROC curve with AUC = 0.74; **(C)** ROC curve with AUC = 0.84; **(D)** ROC curve with AUC = 0.70.

The prognostic potential of the ENO1 protein was assessed through ROC curve analysis (**Figures 3A-D**), using the HS of samples from the benign and malignant groups (**Figure 3A**); benign and malignant with alive outcomes (**Figure 3B**); benign and malignant with death outcomes (**Figure 3C**); and malignant with alive outcomes; and death outcomes (**Figure 3D**). Based on the area under the curve (AUC) values obtained, which are considered to have good accuracy, it was concluded that all the curves had good accuracy (AUC greater than or equal to 0.7). Our results highlight the ROC curve for the malignant with alive outcomes and death outcomes with AUC of 0.70, and the ROC curve for the benign and malignant groups with a death outcome, which obtained an AUC of 0.84, suggesting strong discriminative capacity.

**Figure 4:**
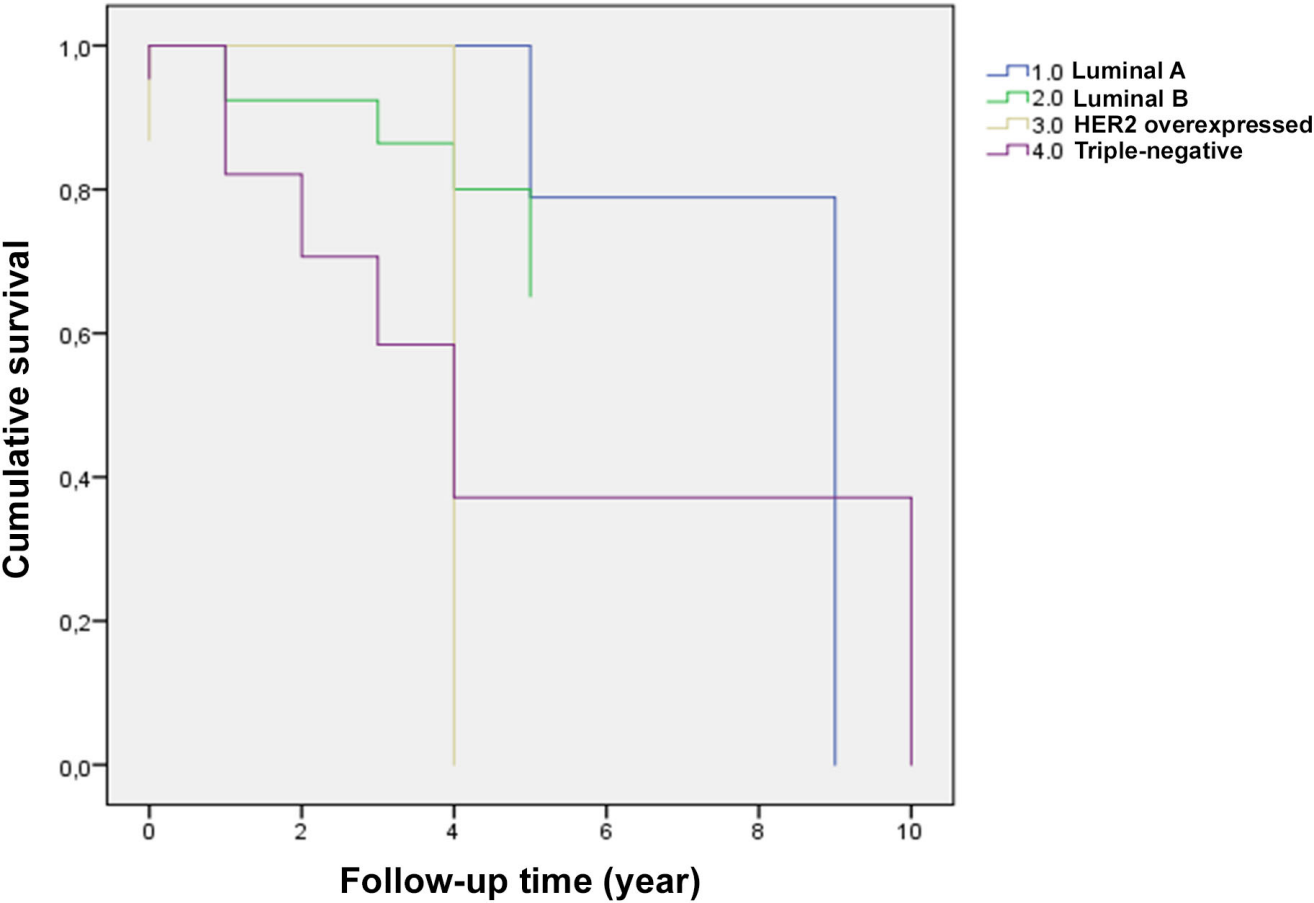
Kaplan Meier graph relating patient survival by molecular subtype of malignant breast tumors. 1.0 – Luminal **(A)** 2 events, 5 censored (71.4% censored); 2.0 - Luminal **(B)** 6 events, 12 censored (66.7% censored); 3.0 - HER2 overexpressed: 3 events, 3 censored (50.0% censored); 4 - Triple-Negative: 7 events, 3 censored (30.0% censored).

The evaluation of COX Regression through the Omnibus Test of Model Coefficients generated a Log Likelihood -2 (value -62.429) and Chi-square = 49.052 (df = 13.604; p < 0.001), indicating that the general model is significant and has good ability to predict the time to the event (**Supplementary Material S3**). When analyzing the ENO1 variable, it was possible to observe a Hazard Ratio (HR) = 1.013 (95% CI: 0.994 to 1.032), suggesting that a one-unit increase in the ENO1 variable increases the risk of the event occurring by 1.3%, but due to the p-value > 0.05 (p = 0.183) it is not considered a significant predictor (**Table 2**). When analyzing the staging variable, a statistical difference was observed with an HR value = 1.454 (95% CI: 1.105 to 1.912) and p = 0.007. This indicates that patients with a more advanced stage have a higher risk of suffering the event (**Table 2**). The survival function plot with mean values of the covariates ENO1 (mean = 88.412) and staging (mean = 3.854) showed that each molecular subtype of breast cancer has a different percentage of censoring, which may suggest that some are more likely to have more censored cases, that is, cases in which the event (death) did not occur. Subtype 1 (luminal A) had high censoring (71.4%), indicating few events for this group, while subtype 4 (triple-negative) had lower censoring (30%); therefore, more events occurred in this subtype (**Figure 4**; **Supplementary Material S3**). Thus, staging is an important variable in prognosis, while ENO1, despite having a positive effect on risk, is not statistically significant. That is, there is insufficient evidence to confirm that ENO1 influences time to event (death). However, a larger sample size or other variables may improve ENO1’s prognostic value. Further studies of ENO1 must be conducted to confirm its role in cancer, more specifically, in breast tumors.

**Table 2:** Data from ENO1 and staging variables analyzed by the omnibus test of model coefficients.

	Coefficient	Standard Error	Wald Statistic	Degree of freedom	P-Value	Hazard Ratio	95.0% CI for HR	
							Lower	Upper
ENO 1	.013	.010	1.769	1	.183	1.013	.994	1.032
Staging	.374	.140	7.160	1	.007	1.454	1.105	1.912

## 4 Discussion

The intensity of immunohistochemical staining of Enolase-1 (ENO1) observed in malignant tumors, especially in cases of unfavorable outcome (death), suggests a direct relationship between the expression of this enzyme and the malignancy of breast tumors (9, 11, 12). Our results showed that ENO1 was detected in both benign and malignant tumors, which was expected as it is an enzyme present throughout the body and essential in the cellular respiration mechanism (8, 14). However, a more intense and heterogeneous staining was observed in malignant tumors, suggesting a possible association between ENO1 overexpression and the aggressive behavior of these neoplasms. Importantly, nuclear staining was detected exclusively in malignant tumors. and higher expression levels correlated with unfavorable clinical outcomes, such as death. These findings suggest that ENO1 expression may be related to tumor malignancy, contributing significantly to diagnosis, especially in identifying more aggressive cases.

The prognostic potential of ENO1 was assessed through ROC curve, demonstrating good diagnostic accuracy (AUC ≥ 0.70) among malignant cases with alive outcome and death, and COX regression analyses. Our results corroborate the study by Giannoudis et al. (2024), which reported ENO1 overexpression in invasive ductal carcinomas, high-grade, advanced, and metastatic tumors, associated with worse survival (17). ENO1 was not considered a significant predictor of prognosis in breast cancer patients. However, staging proved to be an important prognostic variable, indicating that patients with more advanced stages are prone to worse clinical outcomes.

From a functional point of view, ENO1 is classified as a moonlighting protein, performing multiple functions depending on its cellular location (18). We can highlight its glycolytic function in the cytoplasm, essential in cellular energy production, but when deregulated, it favors tumor proliferation and survival, as well as its critical extracellular and nuclear roles (19, 20). When located on the cell surface, it acts as a plasminogen receptor, facilitating tumor invasion and angiogenesis. In the nucleus, it performs regulatory functions in transcription and translation processes, while in the cytoplasm, it contributes to mitochondrial membrane stability and intracellular signaling (18, 21). In its extracellular state, ENO1 can be found associated with extracellular vesicles (EVs) or as a soluble protein, promoting intercellular communication and contributing to tumor progression (12, 13).

In the present study, the nuclear staining of ENO1 was observed in 16 malignant tumor samples, 9 of which belonged to patients with a good prognosis and 7 of which had a death outcome. According to the study of Czogalla et al. (2021), the nuclear staining of ENO1 is associated with its isoform *c-Myc* promoter-binding protein 1 (MBP-1), located in the nucleus and responsible for inhibiting the transcription of *c-Myc*, a tumor growth promoter gene (20). In turn, Lo Presti et al. (2010) analyzed the expression of ENO1 and its MBP-1 isoform in normal breast epithelium and IDC, and observed that cytoplasmic ENO-1 was present in 98% of the tumors analyzed, compared to normal tissues (22). Nuclear MBP-1 was consistently detected in normal tissues, but was also expressed in 35% of IDC tumors, where its presence was associated with longer patient survival. The loss of nuclear MBP-1 expression in IDCs therefore appears to represent a critical event in breast cancer development and progression (22). These results reinforce the need for further studies into the characteristics and functions of this isoform to confirm its potential as a predictor of favorable outcomes.

Recent studies corroborate the multifaceted role of ENO1 in cancer. Qiao et al. (2021) highlight the importance of ENO1 subcellular relocation in cancer pathogenesis and inflammatory processes, associating these changes with increased tumor aggressiveness (21). Huang et al. (2022) also reinforce the association of ENO1 with reduced survival in patients, especially due to its role in glycolytic regulation and its presence in EVs (12). Studies such as that of Alagundagi et al. (2023) pointed to the overexpression of ENO1 in molecular subtypes of breast cancer, linking this characteristic to worse prognosis and clinical outcomes (19). The relevance of ENO1 in cancer goes beyond its diagnostic and prognostic value, encompassing important therapeutic perspectives. In breast cancer, overexpression of ENO1 may be associated with lower metastasis-free survival (17, 23), and poor prognosis, whereas its silencing suppresses proliferation and autophagy, while inducing apoptosis in pancreatic cells (24). Moreover, its low expression has been associated with increased radiosensitivity (25). In pancreatic cancer, autoantibodies against ENO1 have been shown to reduce tumor growth and metastasis (26). More recently, Shen et al. (2025) identified ENO1 as a novel therapeutic target capable of enhancing antitumor immunity and improving clinical outcomes in breast cancer patients (27), indicating that ENO1 may be a promising therapeutic target.

However, despite all the research conducted and the facts analyzed above, ENO1 still presents important limitations that need to be considered. One of the main challenges was the difficulty in associating ENO1 expression with treatment regimens, as complete treatment documentation - including information on drug resistance - was unavailable in most patient records. These patients underwent diagnostic procedures and consultations at our institution but received treatment elsewhere. Another limitation was the similarity observed across three ROC curve analyses (**Figure 3**), which may have reduced the model’s discriminatory power. Finally, the lack of statistically significant findings in the prognostic analysis highlights the need for further, more comprehensive studies to better understand ENO1’s clinical and biological relevance.

Therefore, ENO1 emerges as a multifunctional and highly adaptable enzyme, playing critical roles in different biological and pathological contexts. The results of this study corroborate its relevance in breast cancer, reinforcing its contribution to understanding tumor progression, as well as exploring new therapeutic interventions that can be applied in clinical practice. In this way, the evaluation of ENO1 expression, including its subcellular location, may represent an important tool for diagnostic, and therapeutic individualization in breast cancer.

## Data Availability

The original contributions presented in the study are included in the article/**Supplementary Material**. Further inquiries can be directed to the corresponding author.
