# Supplementary material for: Immunohistochemical analysis of Enolase-1 sublocalization in benign and malignant breast tumors: potential implications for tumor progression and prognosis
Source: Front Oncol. 2025 Oct 16;15:1636394. doi: 10.3389/fonc.2025.1636394 (PMC12571633; doi:10.3389/fonc.2025.1636394)
Supplement: Supplementary file 1 [file Table1.docx]

**Supplementary material**

**Supplementary 1. ENO1 labeling (histoscore) and KI-67 data from malignant breast cancer patients were analyzed retrospectively from 2013 to 2018.**

| **ID** | **HISTOSCORE** | **KI-67** |
| --- | --- | --- |
| 1.1 | 95.4167 | <10% |
| 2.1 | 88.7206 | 20% a 25% |
| 3.1 | 96.7822 | <10% |
| 4.1 | 91.5188 | 9% |
| 5.1 | 73.6743 | 10% a 25% |
| 6.1 | 86.6066 | <10% |
| 7.1 | 86.9978 | 10% a 15% |
| 8.2 | 146.3258 | >30% |
| 9.2 | 78.8089 | >30% |
| 10.2 | 42.3264 | >30% |
| 11.2 | 98.764 | 50% |
| 12.2 | 62.6363 | 20% |
| 13.2 | 79.8502 | 20% |
| 14.2 | 116.0637 | 10% a 25% |
| 15.2 | 99.6945 | 30% |
| 16.2 | 50.33622 | 40% a 50% |
| 17.2 | 65.4485 | >25% |
| 18.2 | 104.0055 | 30% |
| 19.2 | 118.2339 | 18% |
| 20.2 | 107.8642 | >25% |
| 21.2 | 104.89196 | 40% a 50% |
| 22.2 | 69.8232 | 40% |
| 23.2 | 67.5771 | 80% a 90% |
| 24.2 | 143.1414 | >25% |
| 25.2 | 145.1700825 | 40% a 50% |
| 26.3 | 60.9861 | 10% a 25% |
| 27.3 | 46.9729 | 40% |
| 28.3 | 63.5255 | 40% |
| 29.3 | 65.8315 | 30% |
| 30.3 | 55.1915 | <10% |
| 31.3 | 103.6907 | >25% |
| 32.4 | 121.3378 | >30% |
| 33.4 | 113.1591 | 95% |
| 34.4 | 95.5023 | 60% |
| 35.4 | 76.4999 | >30% |
| 36.4 | 121.0221 | >30% |
| 37.4 | 39.9331 | 90% |
| 38.4 | 93.4637 | 30% |
| 39.4 | 142.5616 | 70% |
| 40.4 | 104.5667 | 50% |
| 41.4 | 96.1282 | >25% |

**Supplementary 2. Raw histoscore (HS) data from breast cancer patients were analyzed in the study from 2013 to 2018.**

| **ID** | **HIGH POSITIVE** | **POSITIVE** | **LOW POSITIVE** | **HISTOSCORE** | **SUBTYPE** |
| --- | --- | --- | --- | --- | --- |
| 1.1 | 0 | 10.4877 | 74.4413 | 95.4167 | lumiA |
| 2.1 | 0 | 17.3832 | 53.9542 | 88.7206 | lumiA |
| 3.1 | 0 | 13.5416 | 69.699 | 96.7822 | lumiA |
| 4.1 | 0 | 8.4431 | 74.6326 | 91.5188 | lumiA |
| 5.1 | 0 | 2.8257 | 68.0229 | 73.6743 | lumiA |
| 6.1 | 0 | 11.1128 | 64.381 | 86.6066 | lumiA |
| 7.1 | 0 | 5.481 | 76.0358 | 86.9978 | lumiA |
| 8.2 | 0 | 56.6389 | 33.048 | 146.3258 | lumiB |
| 9.2 | 0 | 20.8321 | 37.1447 | 78.8089 | lumiB |
| 10.2 | 0 | 2.0778 | 38.1908 | 42.3464 | lumiB |
| 11.2 | 0 | 2.2472 | 93.582 | 98.0764 | lumiB |
| 12.2 | 0 | 1.224 | 60.1883 | 62.6363 | lumiB |
| 13.2 | 0 | 13.5335 | 52.7832 | 79.8502 | lumiB |
| 14.2 | 0 | 40.5465 | 34.9707 | 116.0637 | lumiB |
| 15.2 | 0 | 22.63 | 54.4345 | 99.6945 | lumiB |
| 16.2 | 0 | 1.19221 | 47.9518 | 50.33622 | lumiB |
| 17.2 | 0 | 1.8668 | 61.7149 | 65.4485 | lumiB |
| 18.2 | 0 | 33.565 | 36.8755 | 104.0055 | lumiB |
| 19.2 | 0 | 19.6981 | 78.8377 | 118.2339 | lumiB |
| 20.2 | 0 | 12.996 | 81.8722 | 107.8642 | lumiB |
| 21.2 | 0 | 16.2368 | 72.41836 | 104.89196 | lumiB |
| 22.2 | 0 | 2.8469 | 64.1294 | 69.8232 | lumiB |
| 23.2 | 0 | 15.1137 | 37.3497 | 67.5771 | lumiB |
| 24.2 | 0 | 46.5521 | 50.0372 | 143.1414 | lumiB |
| 25.2 | 0 | 50.8611 | 43.4475 | 145.1700825 | lumiB |
| 26.3 | 0 | 10.2467 | 40.4927 | 60.9861 | HER2 |
| 27.3 | 0 | 7.5487 | 31.8755 | 46.9729 | HER2 |
| 28.3 | 0 | 5.6998 | 52.1259 | 63.5255 | HER2 |
| 29.3 | 0 | 3.8828 | 58.0659 | 65.8315 | HER2 |
| 30.3 | 0 | 2.3814 | 50.4287 | 55.1915 | HER2 |
| 31.3 | 0 | 6.3026 | 91.0855 | 103.6907 | HER2 |
| 32.4 | 0 | 31.8603 | 57.6172 | 121.3378 | triple-neg |
| 33.4 | 0 | 33.8386 | 45.4819 | 113.1591 | triple-neg |
| 34.4 | 0 | 19.2296 | 57.0431 | 95.5023 | triple-neg |
| 35.4 | 0 | 15.8703 | 44.7593 | 76.4999 | triple-neg |
| 36.4 | 0 | 23.4113 | 74.1995 | 121.0221 | triple-neg |
| 37.4 | 0 | 2.2612 | 35.4107 | 39.9331 | triple-neg |
| 38.4 | 0 | 6.2726 | 80.9185 | 93.4637 | triple-neg |
| 39.4 | 0 | 44.8853 | 52.791 | 142.5616 | triple-neg |
| 40.4 | 0 | 7.7354 | 89.0959 | 104.5667 | triple-neg |
| 41.4 | 0 | 23.46 | 49.2082 | 96.1282 | triple-neg |
| 42.5 | 0 | 28.7493 | 41.2199 | 98.7185 | fibroad |
| 43.5 | 0 | 2.3536 | 34.3432 | 39.0504 | fibroad |
| 44.5 | 0 | 31.1327 | 38.8198 | 101.0852 | fibroad |
| 45.5 | 0 | 17.013 | 30.2054 | 64.2314 | fibroad |
| 46.5 | 0 | 0.184 | 32.2435 | 32.6115 | fibroad |
| 47.5 | 0 | 0.3258 | 41.4088 | 42.0604 | fibroad |
| 48.5 | 0 | 0.4187 | 26.1315 | 26.9689 | fibroad |

**Supplementary 3. Data processing to perform COX regression using the SPSS program (IBM, version 24, 2014).**

| **Case Processing Summary** | | | |
| --- | --- | --- | --- |
|  | | N | Percentage |
| Available cases under analysis | Event^a^ | 18 | 43.9% |
|  | Censored | 23 | 56.1% |
|  | Total | 41 | 100.0% |
| Discarded cases | Cases with missing values | 0 | 0.0% |
|  | Cases with negative time | 0 | 0.0% |
|  | Cases censored before the first event in a layer | 0 | 0.0% |
|  | Total | 0 | 0.0% |
| Total | | 41 | 100.0% |
| a. Dependent Variable: FOLLOW-UP TIME | | | |
|  |  |  |  |
| Layer status^a^ | | | |
| Layer | Event | Censored | Censored percentage |
| 1.0 (luminal A) | 2 | 5 | 71,4% |
| 2.0 (luminal B) | 6 | 12 | 66,7% |
| 3.0 (HER2 overexpressed) | 3 | 3 | 50,0% |
| 4.0 (triple-negative) | 7 | 3 | 30,0% |
| Total | 18 | 23 | 56,1% |
| a. The stratum variable is: SUBTYPE | | | |
| **Omnibus Tests of Model Coefficients** |  |  |  |
| Log da Verossimilhança -2 |  |  |  |
| 62,429 |  |  |  |

**Omnibus Tests of Model Coefficients^a^**

| Log Likelihood -2 | **Overall (score)** | | | **change of the previous step** | | | **change of the previous block** | | |
| --- | --- | --- | --- | --- | --- | --- | --- | --- | --- |
|  | **chi-square** | **gl** | **Sig.** | **chi-square** | **gl** | **Sig.** | **chi-square** | **gl** | **Sig.** |
| **49.052** | **13.604** | **2** | **.001** | **13.377** | **2** | **.001** | **13.377** | **2** | **.001** |

1. **Starting Block Number 1. Method = Enter**

| **Covariate means** | |
| --- | --- |
|  | mean |
| ENO1 | 88.412 |
| Staging | 3.854 |
